# Imaging the metabolic reprograming of fatty acid synthesis pathway enables new diagnostic and therapeutic opportunity for breast cancer

**DOI:** 10.1186/s12935-023-02908-8

**Published:** 2023-04-29

**Authors:** Fukai Wang, Shuangshuang Ma, Panpan Chen, Yuhao Han, Zhaoyun Liu, Xinzhao Wang, Chenglong Sun, Zhiyong Yu

**Affiliations:** 1 Breast Cancer Center, Shandong Cancer Hospital and Institute, Shandong First Medical University, Shandong Academy of Medical Sciences, Jinan, 250117 China; 2 Shandong Provincial Key Laboratory of Radiation Oncology, Cancer Research Center, Shandong Cancer Hospital and Institute, Shandong First Medical University and Shandong Academy of Medical Sciences, Jinan, 250117 China; 3 School of Pharmaceutical Sciences, Qilu University of Technology (Shandong Academy of Sciences), Jinan, 250014 China; 4 Key Laboratory for Applied Technology of Sophisticated Analytical Instruments of Shandong Province, Shandong Analysis and Test Center, Qilu University of Technology (Shandong Academy of Sciences), Jinan, 250014 China

**Keywords:** Mass spectrometry imaging, Metabolic reprograming, Breast cancer, Fatty acid synthesis pathway, Diagnosis and therapy

## Abstract

**Background:**

Reprogrammed metabolic network is a key hallmark of cancer. Profiling cancer metabolic alterations with spatial signatures not only provides clues for understanding cancer biochemical heterogeneity, but also helps to decipher the possible roles of metabolic reprogramming in cancer development.

**Methods:**

Matrix-assisted laser desorption/ionization mass spectrometry imaging (MALDI-MSI) technique was used to characterize the expressions of fatty acids in breast cancer tissues. Specific immunofluorescence staining was further carried out to investigate the expressions of fatty acid synthesis-related enzymes.

**Results:**

The distributions of 23 fatty acids in breast cancer tissues have been mapped, and the levels of most fatty acids in cancer tissues are significantly higher than those in adjacent normal tissues. Two metabolic enzymes, fatty acid synthase (FASN) and acetyl CoA carboxylase (ACC), which being involved in the *de novo* synthesis of fatty acid were found to be up-regulated in breast cancer. Targeting the up-regulation of FASN and ACC is an effective approach to limiting the growth, proliferation, and metastasis of breast cancer cells.

**Conclusions:**

These spatially resolved findings enhance our understanding of cancer metabolic reprogramming and give an insight into the exploration of metabolic vulnerabilities for better cancer treatment.

**Supplementary Information:**

The online version contains supplementary material available at 10.1186/s12935-023-02908-8.

## Background

Breast cancer has replaced lung cancer as the world’s leading cancer, with 2.26 million new cases worldwide in 2020 [1]. It also ranks first among new cases and deaths of female cancer [2]. A significant hallmark of tumor cells is that they can reprogram their metabolic networks to support the synthesis of macromolecules and energy compounds being needed for tumor growth, proliferation, and metastasis [3, 4]. The metabolic reprogramming of breast cancer has also been thoroughly investigated by many researchers. For example, breast cancer cells exhibit typical features of warburg effect. They transfer the glucose metabolism pathway from mitochondrial oxidative phosphorylation to aerobic glycolysis for rapid energy generation [5, 6]. Metabolic enzymes, being related to glucose transmembrane transport and glycolysis such as glucose transporter 1 (GLUT1) and hexokinase 2 (HK2) are highly expressed in breast cancer cells [7, 8]. And compared with normal cells, breast cancer cells usually present lower levels of glucose and higher levels of lactic acid [9]. In addition, breast cancer cells also display strong addiction to glutamine. There is growing evidence that glutaminase (GLS), a key enzyme that regulates the metabolism of glutamine, is significantly up-regulated in breast cancer cells, resulting in excessive utilization of glutamine in breast cancer cells [10, 11]. In previous studies, we found that carnitine and carnitine system-mediated fatty acid β-oxidation pathway were significantly reprogrammed in breast cancer tissues [12]. L-carnitine, short-chain acylcarnitines, and three carnitine-mediated enzymes: carnitine palmitoyltransferase 1 A (CPT1A), carnitine palmitoyltransferase 2 (CPT2), and carnitine acetyltransferase (CRAT) were all up-regulated in breast cancer tissues.

Fatty acids are important building blocks of cell phospholipids and glycolipids. The *de novo* synthesis of fatty acids is an indispensable metabolic pathway that converts nutrients into metabolic intermediates for cell membrane generation, energy metabolism, and signal transductions [13, 14]. Emerging work indicates that the alteration of fatty acid synthesis pathway is closely related to the occurrence, development, and metastasis of breast cancer [15–19]. Park et al. found that the blood level of docosahexaenoic acid in breast cancer patients was significantly lower than that in healthy controls, and docosahexaenoic acid could be used as a potential diagnostic marker for breast cancer [20]. Xu et al. showed that fatty acid synthase could facilitate the migration of breast cancer cells by regulating changes in fatty acid metabolism [18]. It also has been reported that the levels of polyunsaturated *n*-3 fatty acids in breast adipose tissues are closely related to the multifocality of breast cancer [21]. However, it should be noted that breast cancer tissues are highly heterogeneous, and it is crucial to accurately characterize the spatial distributions of fatty acids in different cancer tissue micro-regions. Mass spectrometry imaging (MSI) is a label free technique that can not only detect the contents of metabolites, but also directly map the spatial distributions of metabolites in biological tissues [22, 23]. Matrix-assisted laser desorption/ionization (MALDI)- and desorption electrospray ionization (DESI)-MSI are currently the most commonly used MSI techniques, and the maximum spatial resolution can reach 1.4 μm [24–27]. They have shown very valuable application prospects in characterizing the spatiotemporal metabolic network of tumors, diagnosing the occurrence of cancers and elucidating the therapeutic effect of antitumor drugs [28–38].

The aim of this study is to image the spatial alterations of fatty acids and their synthetic pathway in heterogeneous breast cancer tissues, and to discover potential metabolic liabilities that can be exploited for cancer therapy. Combining spatially resolved MALDI-MSI and immunofluorescence technique, we found that the expressions of fatty acids and their synthesis-related enzymes fatty acid synthase (FASN) and acetyl CoA carboxylase (ACC) in breast cancer tissues were significantly higher than those in adjacent normal tissues. A multivariate statistical classification model constructed based on the spatial expressions of fatty acids could accurately identify breast cancer tissues and adjacent normal tissues. Furthermore, we found that the targeted inhibition of the up-regulated FASN and ACC was an effective approach to limit the growth, proliferation, and metastasis of breast cancer cells. Exploring the spatial signatures and alterations of fatty acids and their *de novo* synthetic pathway in heterogeneous cancer tissues can yield important insights into how to target fatty acid metabolism.

## Methods

### Human breast cancer tissue sample collection

All experiments were approved by the Institutional Ethical Review Board of Shandong Cancer Hospital and Institute (No. 201,907,005). 60 cases of breast cancer tissue specimens were obtained through surgery. Patient characteristics are listed in Table S1. The postoperative breast cancer tissue specimens include cancer regions and adjacent normal regions. Before MALDI-MSI experiments, all the collected tissue specimens were stored at -80 °C refrigerator.

### Tissue sample processing

Five sets of 10 μm thick adjacent frozen tissue sections were prepared at -25 °C using a NX50 NOVPD cryostat (Thermo Fisher Scientific). One of the tissue sections was used for H&E staining. The other two tissue sections were used for MALDI-MSI analysis. The last two tissue sections were used for immunofluorescence staining. The tissue sections used for H&E and immunofluorescence staining were thaw-mounted on superfrost™ plus microscope slides. The tissue sections used for MALDI-MSI analysis need to be thaw-mounted on indium tin oxide-coated slides.

### Matrix coating and MALDI-MSI analysis

Before spraying the matrix, the tissue sections on the slides were dried in vacuum for 15 min. To detect more fatty acids, we compared the MALDI-MSI performance of fatty acids with 9-AA, 1,5-DAN, and BNDM as matrix respectively. Eight passes of 9-AA (10.0 mg/mL), eight passes of 1,5-DAN (2.0 mg/mL), and twelve passes of BNDM (1.0 mg/mL) were sprayed on three adjacent tissue sections using a HTX TM-Sprayer™. The flow rate of matrix solution, nozzle temperature, and spray pressure were set to 0.075 mL/min, 55 °C, and 10 psi, severally. Track spacing was 3 mm, and track speed was 80 cm/min. After spraying the matrix, we used MALDI tissuetyper™ TOF/TOF mass spectrometer (Bruker Daltonics) to conduct MALDI-MSI experiments. Mass spectra were collected in the range of *m/z* 80 ~ 1000 in negative ion mode. The spatial resolution was 100 μm. Smartbeam™ 3D laser was emitted at a repetition rate of 5000 Hz.

### Immunohistochemistry

To explore the spatial expressions of FASN and ACC in breast cancer tissue sections, two sets of tissue sections were subjected to immunofluorescence analysis. After fixed in 4% paraformaldehyde solution for 15 min, the breast cancer tissue sections were incubated with 1% bovine serum albumin (BSA) for 30 min. Then, these tissue sections were incubated with FASN antibody (Abcam Cat# ab128870, RRID: AB_11143436, 1:200) and ACC antibody (Abcam Cat# ab109368, RRID: AB_10864809, 1:100) overnight in a 4 °C incubator. Next, the tissue section was incubated with secondary antibody for 50 min, and then the nucleus were counterstained with DAPI. The tissue sections were added AutoFluo Quencher for another 5 min. Finally, the slices were placed under a scanner (3DHISTECH) to take images.

### Cell culture and treatment

Human breast cancer cell line MDA-MB-231 was purchased from the Cell Bank of Shanghai Institute of Biochemistry and Cell Biology (Chinese Academy of Sciences). Cells were cultured in dulbecco’s modified eagle medium (DMEM) (KeyGen Biotech) supplemented with 10% fetal bovine serum (FBS) (Gibco) and 100 U/mL antibiotics (penicillin and streptomycin) at 37 °C in a humidified incubator with 5% CO_2_. When the cell confluence reached 80–90%, the TVB-2640 (Selleck, S9714) and/or PF-05175157 (Selleck, S6672) were added respectively. After 24 h, the cell viability was detected by CCK-8 assay and colony formation test, the cell proliferation was reflected with EdU Staining and DAPI nuclear staining, and the cell migration was monitored by wound healing assay.

### Data analysis

MALDI-MS images were constructed and processed using SCiLS Lab 2018b software (SCiLS GmbH). Region-specific fatty acid profiles were extracted in SCiLS Lab. Principal component analysis, orthogonal partial least squares discrimination analysis, partial least squares discrimination analysis, and statistical prediction were performed on SIMCA-P software package (Version 14.0, Umetrics AB). Receiver operating characteristic curve analysis and t-test were carried out on GraphPad Prism software (Version 6.0). Immunofluorescence images were processed by CaseViewer software (3D Histech).

## Results

### Development of a high-coverage mass spectrometry imaging method to map the spatial distributions of fatty acids in breast cancer tissues

**Fig. 1:**
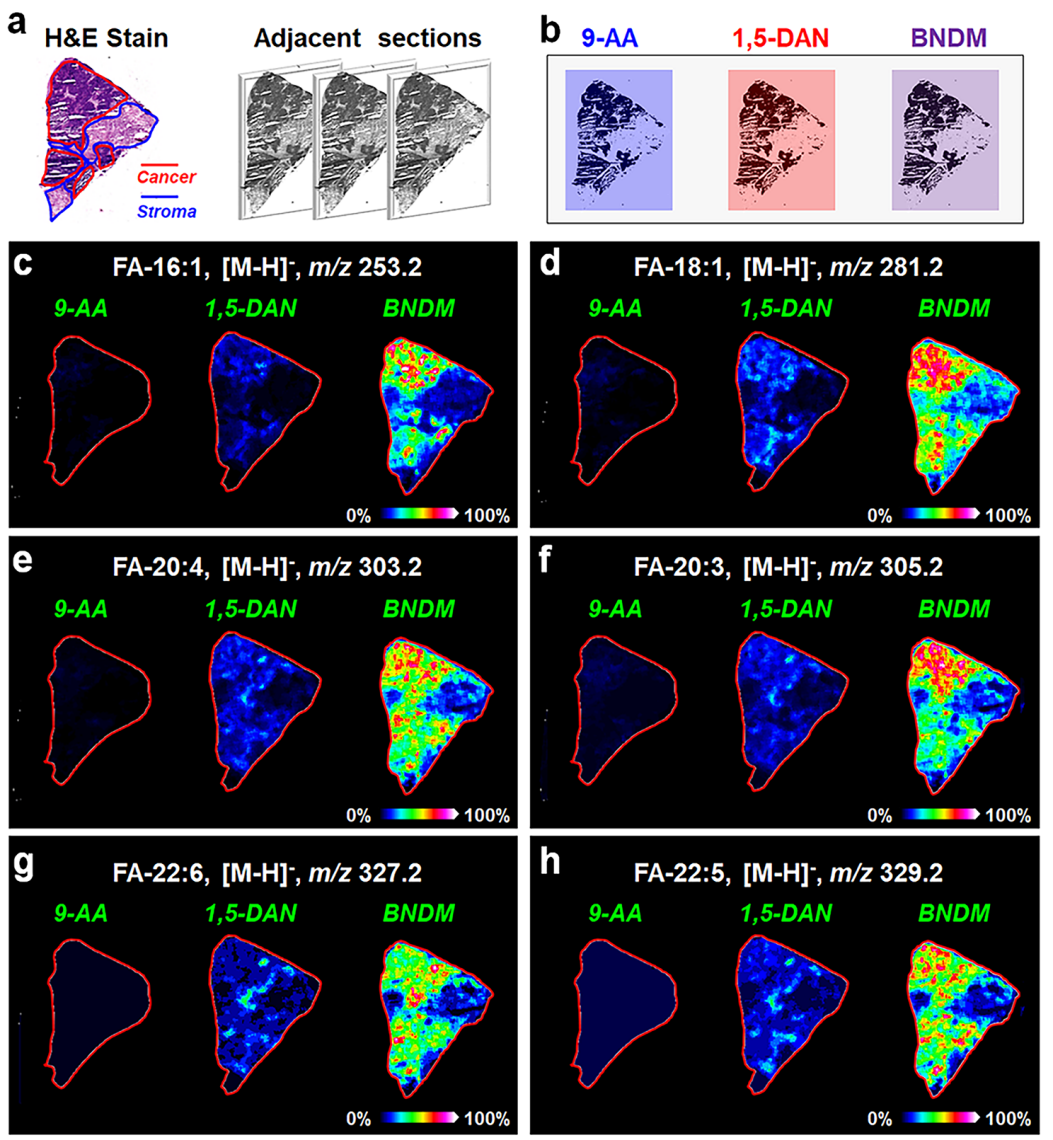
**(a)** H&E stain and optical image of breast cancer tissue section. **(b)** Spray 9-AA, 1,5-DAN, and BNDM on adjacent breast cancer tissue sections. **(c-h)** MS images of representative fatty acids (FA) in breast cancer tissue sections using 9-AA, 1,5-DAN, and BNDM as MALDI matrices

The postoperative breast cancer tissues were cut into continuous tissue sections, and one of them was used to perform H&E staining. As shown in Fig. 1a, the postoperative breast cancer tissue section contains not only cancer areas, but also normal areas adjacent to the cancer areas. As an important component of cell architecture, fatty acids also play a crucial role in cell signal transduction and energy metabolism. Mapping the spatial distributions of fatty acids in heterogeneous breast cancer tissues is significant for our understanding of the fatty acid biosynthesis and metabolism.

MALDI-MSI offers an insight approach to visualizing the spatial distributions of metabolites in heterogeneous biological tissues. However, the selection of MALDI matrix is a key factor for metabolite imaging. Considering that the fatty acid contains carboxyl group in their structure, it is more suitable to perform MALDI-MSI analysis in negative ion detection mode. 9-Aminoacridine (9-AA) and 1,5-diaminonaphthalene (1,5-DAN) are two commonly used MALDI matrices in negative ion mode. 1,1’-Binaphthyl-2,2’-diamine (BNDM) is a dual-polarity matrix developed by our group, which can facilitate the detection of 301 negative and 175 positive metabolite ions in biological tissues [39]. Here, we compared the mass spectrometry imaging performance of fatty acids in breast cancer tissue sections using 9-AA, 1,5-DAN, and BNDM as matrix respectively (Fig. 1b). The MS images of representative fatty acids (FAs) such as FA-16:1 ([M-H]^−^, *m/z* 253.2), FA-18:1 ([M-H]^−^, *m/z* 281.2), FA-20:4 ([M-H]^−^, *m/z* 303.2), FA-20:3 ([M-H]^−^, *m/z* 305.2), FA-22:6 ([M-H]^−^, *m/z* 327.2), and FA-22:5 ([M-H]^−^, *m/z* 329.2) in breast cancer tissue sections were illustrated in Fig. 1c-Fig. 1h. When 9-AA was used as MALDI matrix, FAs exhibited very weak MS signals. Although many FAs could be detected and imaged with 1,5-DAN as the MALDI matrix, the MS signal of FAs were significantly enhanced when BNDM was used as the matrix. In view of this, we chose BNDM as the matrix for MALDI-MSI of FAs in breast cancer tissues. And when BNDM was used as the MALDI matrix, a total of 23 FAs, including one C_14_ FA (FA-14:1), three C_16_ FAs (FA-16:0, FA-16:1, FA-16:2), four C_18_ FAs (FA-18:0, FA-18:1, FA-18:2, FA-18:3), six C_20_ FAs (FA-20:0, FA-20:1, FA-20:2, FA-20:3, FA-20:4, FA-20:5), seven C_22_ FAs (FA-22:0, FA-22:1, FA-22:2, FA-22:3, FA-22:4, FA-22:5, FA-22:6), and two C_24_ FAs (FA-24:0, FA-24:1) were successfully imaged in breast cancer tissues (Fig. S1). To our knowledge, this is the mass spectrometry imaging method detecting the largest number of FAs in biological tissues.

### The expressions of FAs were significantly up-regulated in breast cancer tissues

**Fig. 2:**
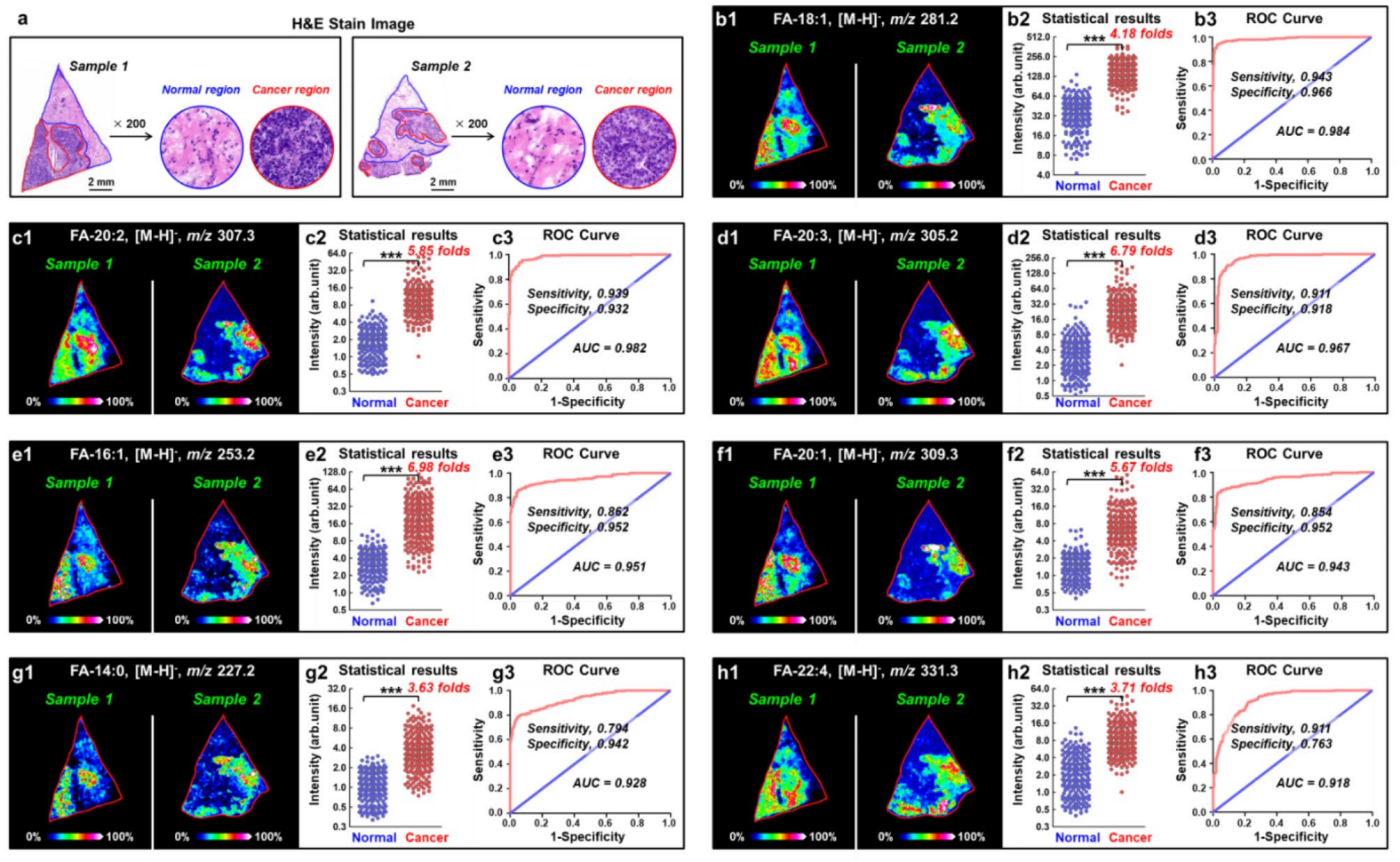
**(a)** H&E stain images of two representative breast cancer tissue sections. **(b-h)** MS images, statistical results and ROC curves of seven representative fatty acids in breast cancer tissues (*n*_normal_, 207; *n*_cancer_, 246).The scale of *y*-axis is log 2; ***, *p* < 0.001

Using the high-coverage MALDI-MSI method, we analyzed the expressions of FAs in the postoperative tissues from 60 breast cancer patients. For most postoperative specimens, they contain both cancer and normal tissues. Figure 2a shows the typical H&E stain images of two breast cancer tissues, and the boundary between cancer areas and normal areas can be accurately distinguished. After performing MALDI-MSI analysis, we extracted 246 cancer region mass spectra and 207 normal region mass spectra from 60 breast cancer tissue samples based on the histological image. The results showed that the expressions of most fatty acids in the cancer region were significantly higher than those in the paired normal region. Figure 2b**-**Fig. 2h demonstrated the MS images and statistical results of the seven most up-regulated FAs in breast cancer region. The levels of FA-20:3 and FA-16:1 in breast cancer region increased nearly 7 times than normal region (Fig. 2d and e). FA-20:2 and FA-20:1 increased by 5.85 times and 5.67 times in the cancer region, separately (Fig. 2c and f). The increase contents of FA-18:1, FA-22:4, and FA-14:0 also reached 4.18, 3.71, and 3.63 times, separately (Fig. 2b, h and g).

Furthermore, receiver operating characteristic (ROC) curve analysis was used to examine the usefulness of FAs in distinguishing breast cancer and normal regions. The performance of ROC curve was evaluated by the area under curve (AUC). ROC curves generated from FA-18:1, FA-20:2, FA-20:3, FA-16:1, FA-20:1, FA-14:0, and FA-22:4 showed good discrimination ability with AUC values of 0.984, 0.982, 0.967, 0.951, 0.928, 0.943, and 0.918, respectively (Fig. 2b-Fig. 2h). These results indicated that the contents of the above seven FAs had high diagnostic value for breast cancer region.

### Molecular identification and diagnosis of breast cancer tissue based on the expression of FAs

**Fig. 3:**
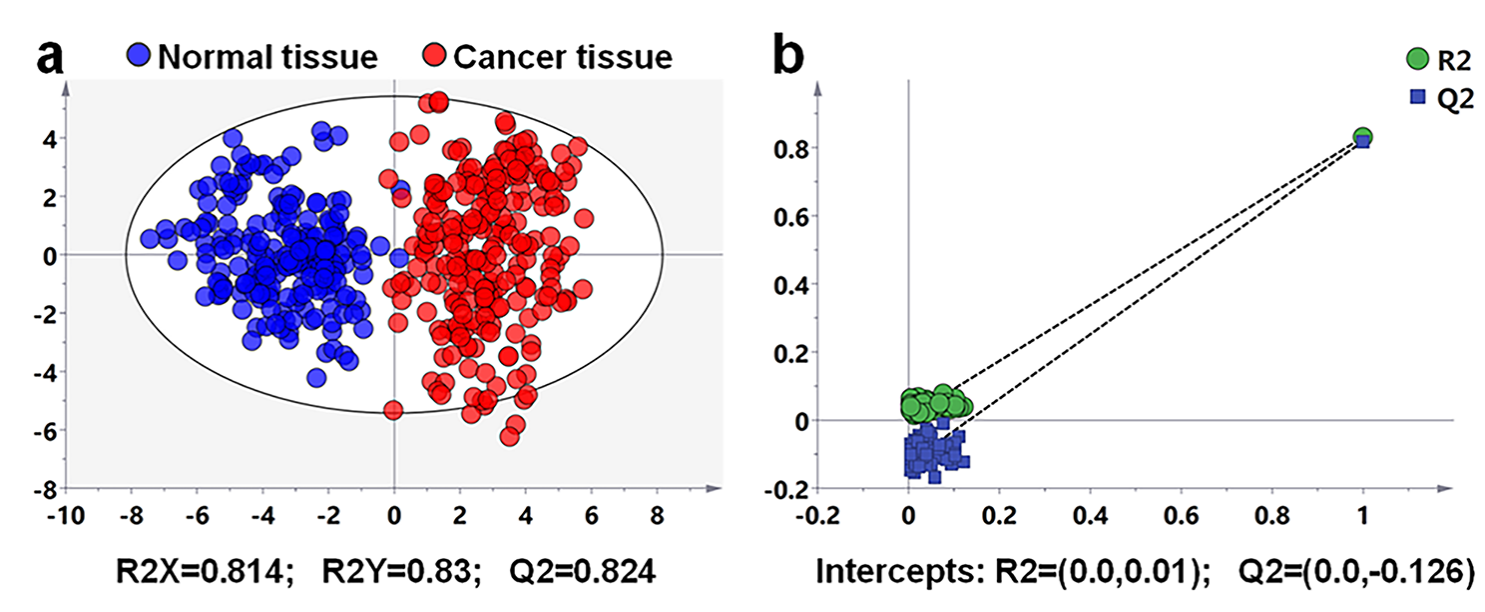
**(a)** OPLS-DA score plots based on MALDI-MSI data of 23 fatty acids in breast cancer and paired normal tissues (*n*_normal_, 207; *n*_cancer_, 246). **(b)** The PLS validation plots obtained from 100 permutation tests

To further explore the potential value of fatty acids in the identification and diagnosis of breast cancer, we carried out multivariate statistical analysis based on the FAs profiles of 246 breast cancer regions and 207 adjacent normal regions. Unsupervised principal component analysis (PCA) was first conducted to investigate the global clustering and grouping trend of breast cancer and adjacent normal regions. The PCA score plots was shown in Fig. S2, although there was some overlap between breast cancer and adjacent normal regions, the grouping trend in cancer and normal regions was very obvious. Furthermore, we carried out supervised orthogonal partial least squares discrimination analysis (OPLS-DA), which could fully reflect the differences between cancer and normal regions by adding artificial grouping information. Figure 3a showed the OPLS-DA model based on the MALDI-MSI data of 23 FAs. The model exhibited very good grouping characteristics, with Q2 (cum) value of 0.824 across one predictive and three orthogonal [1 + 3] components, R2 (X) value of 0.814, and R2 (Y) value of 0.83. Then, we carried out a random permutation test with partial least squares discrimination analysis (PLS-DA) to evaluate the validity of this OPLS-DA model. The permutation test model was produced with intercepts of Q2 = -0.126 and R2 = 0.01, indicating that the OPLS-DA did not overfitting (Fig. 3b).

**Fig. 4:**
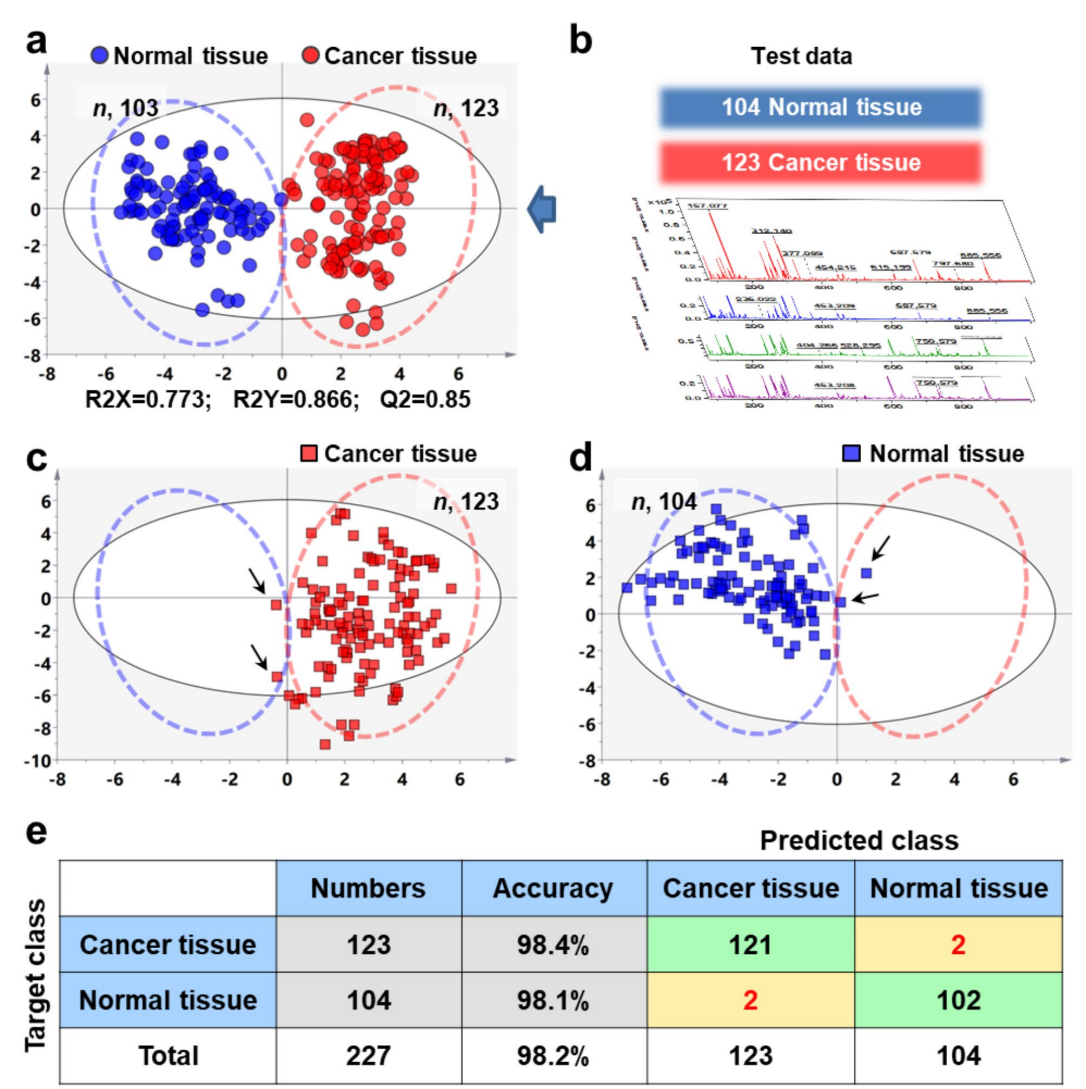
**Statistical prediction of breast cancer based on OPLS-DA classification model. (a)** OPLS-DA score plots based on MALDI-MSI data of 23 fatty acids in breast cancer and normal tissues of batch (1) **(b)** MALDI-MS profiles of 23 fatty acids in breast cancer and normal tissues of batch (2) **(c)** Predicted results of breast cancer tissues from batch 2. **(d)** Predicted results of breast normal tissues from batch 2. **(e)** The confusion matrix of the OPLS-DA model showing classification results of BC tissues from batch 2

Next, we built an OPLS-DA classification model to identify the category of samples based on the expressions of FAs. 246 cancer region mass spectra and 207 normal region mass spectra were randomly divided into two batches. The first batch included 123 cancer region mass spectra and 103 normal region mass spectra, and the second batch contained 123 cancer region mass spectra and 104 normal region mass spectra. The data from the first batch were trained to build OPLS-DA model. As shown in Fig. 4a, the OPLS-DA model showed good clustering and grouping trend with Q2 (cum) value of 0.85, R2 (X) value of 0.773, and R2 (Y) value of 0.866. Then, the samples from second batch were taken as unknown samples and brought into the OPLD-DA model to perform classification analysis (Fig. 4b). Figure 4c**-**Fig. 4e illustrated the predicted results on the second batch of samples. A total of two tumor regions were incorrectly identified as normal regions (arrows in Fig. 4c), and two normal samples were misjudged as cancer samples (arrows in Fig. 4d). The overall classification accuracy for all these 227 cases of samples (123 cancer samples and 104 normal samples) reached 98.2% (Fig. 4e).

### Imaging and correlating the spatial features of fatty acids and fatty acid synthesis-related metabolic enzymes in breast cancer tissues

**Fig. 5:**
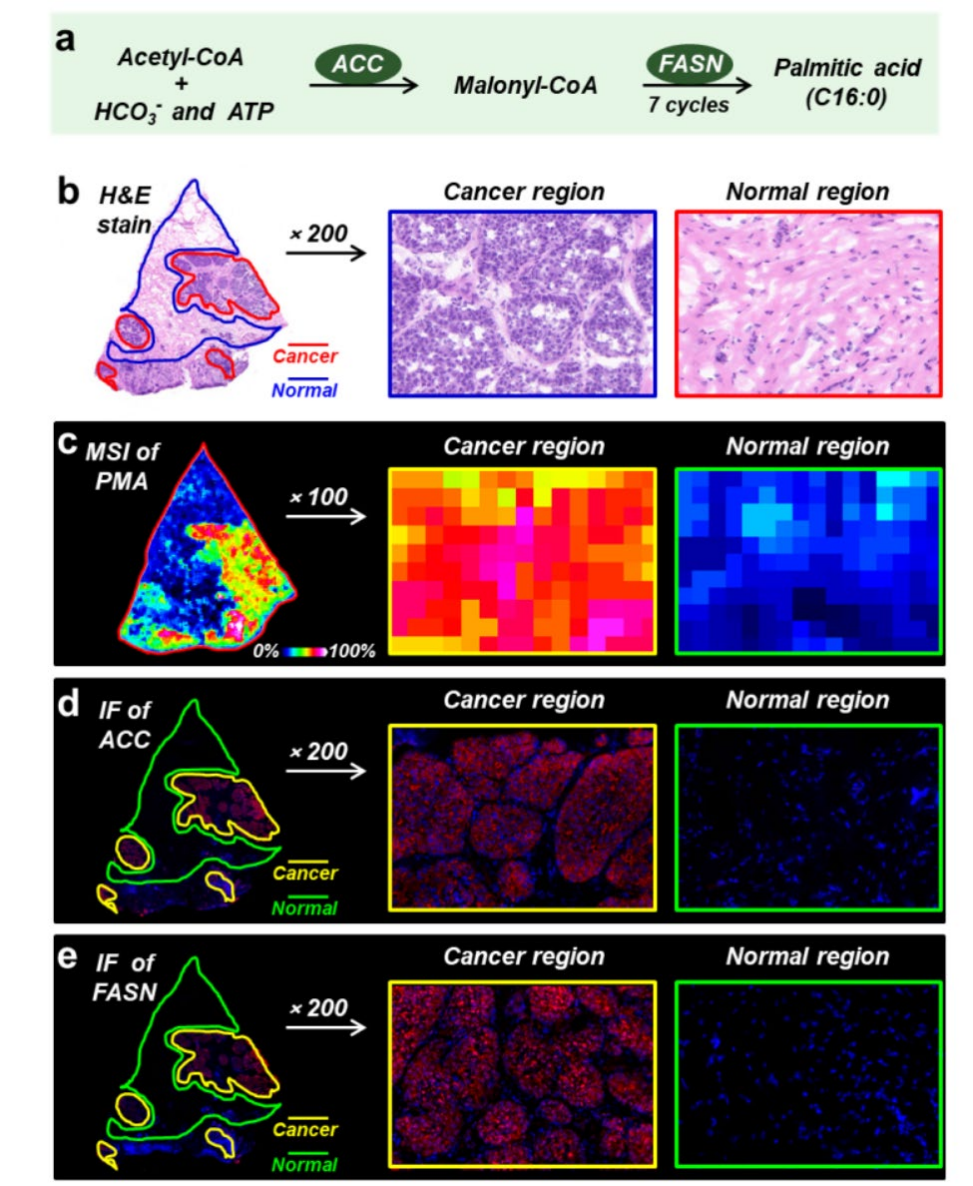
**(a)** The biosynthetic pathway of fatty acids. **(b)** The H&E stain image of breast cancer tissue section. **(c)** MS image of PMA in breast cancer tissue section. **(d-e)** Immunofluorescence images of ACC and FASN in breast cancer tissue section, ACC and FASN are red staining, blue is DAPI staining. ACC, acetyl-CoA carboxylase; FASN, fatty acid synthase; PMA, palmitic acid

In this study, we found that the expressions of most fatty acids in breast cancer region were significantly higher than those in adjacent normal region. Generally, cancer cells tent to improve the biosynthesis of endogenous metabolites to meet their infinite proliferation. From the biosynthesis pathway, the *de novo* synthesis of fatty acids requires two core steps (Fig. 5a): (*i*) ACC catalyzes the formation of malonyl-CoA from acetyl-CoA; (*ii*) after 7 cycles of priming, loading, condensation, reduction, dehydration, reduction, and hydrolysis, one molecule of acetyl-CoA and seven molecules of malonyl-CoA are continuously condensed into palmitic acid under the catalysis of FASN. Palmitic acid (PMA, FA-16:0) is the first fatty acid synthesized *de novo*, and it can continue to be desaturated and elongated into several types of fatty acids with different saturations and lengths.

Palmitic acid showed much stronger ion intensity in the cancer region than the adjacent normal region in breast cancer tissue sections (Fig. 5b and c). ACC and FASN are two rate-limiting enzymes for palmitic acid synthesis. We speculated whether the up-regulation of palmitic acid and other fatty acids in breast cancer region was caused by the abnormal expression of ACC and FASN. Therefore, we further analyzed the spatial expressions of ACC and FASN in breast cancer tissues. To match the spatial characteristics of fatty acids and related metabolic enzymes, we performed immunofluorescence (IF) analysis on adjacent tissue sections. Figure 5d and e illustrated the IF stain images of ACC and FASN in adjacent breast cancer tissue sections. Significantly, ACC and FASN all exhibited stronger expression in the cancer region than adjacent normal region, which were highly consistent with the spatial characteristics of palmitic acid.

### Exploring the biological functions of ACC and FASN in the proliferation and metastasis of breast cancer cells

By performing spatially resolved MALDI-MSI and IF analysis, we found that fatty acids and fatty acid synthesis-related metabolic enzymes all were up-regulated in breast cancer regions. Further, we explored the roles of ACC and FASN, two key enzymes in the fatty acid synthesis pathway, in the proliferation and metastasis of breast cancer cells.

**Fig. 6:**
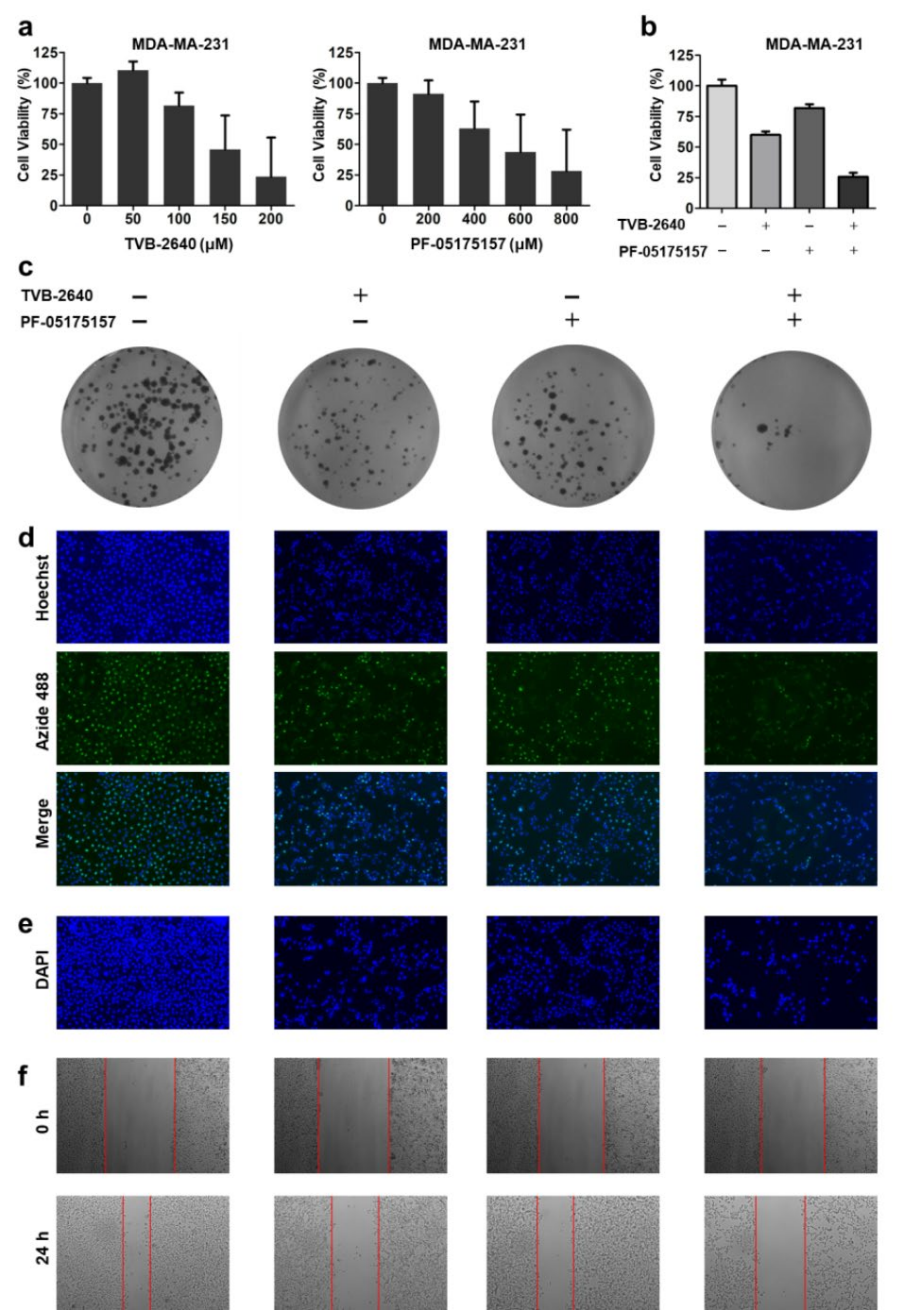
**The effect of ACC and FASN inhibitor on MDA-MB-231 cells. (a-b)** Cell viability was determined by the CCK-8 assay. **(c)** Colony formation was assessed by the colony formation assay. **(d)** EdU-488 detects the DNA synthesis in MDA-MB-231 cells. **(e)** DAPI staining reflects the change of nucleus. **(f)** The migration of TNBC cells was quantified by wound healing

Previous studies have shown that TVB-2640 and PF-05175157 can inhibit the expression of FASN and ACC in tumor cells, respectively. Here, TVB-2640 and PF-05175157 were used to treat human triple-negative breast cancer MDA-MB-231 cell. To observe the toxic effects of TVB-2640 and PF-05175157, we set different concentrations of two inhibitors to treat MDA-MB-231 cells. The 24 h IC_50_ of TVB-2640 and PF-05175157 to MDA-MB-231 cells are 131.8 µM and 428.5 µM severally. TVB-2640 and PF-05175157 inhibited the activity of triple-negative breast cancer cells in a concentration-dependent manner (Fig. 6a). To further observe the effect of the two inhibitors on MDA-MB-231 cells, these cells were divided into four groups including DMSO, TVB-2640 (100 µM), PF-05175157 (300 µM) and the combination group. The CCK8 experiment showed that the cell activity of the combination group was significantly lower than that of the single-inhibitor groups (Fig. 6b). The clone formation experiment found that the number of cell colonies in the single-inhibitor groups were significantly lower than that in the control group, and the number of cell colonies was further lower in the combination group compared with the single-inhibitor groups (Fig. 6c). 5-Ethynyl-2’-deoxyuridine (EdU) can replace thymidine in the synthesis of DNA. EdU-488 can reflect the cell proliferation by detecting the synthesis of DNA. Our results showed that TVB-2640 and PF-05175157 could inhibit the DNA synthesis in breast cancer cells compared with the control group, and the effect of the combination group was significantly better than the single-inhibitor groups (Fig. 6d). In addition, the impacts of TVB-2640 and PF-05175157 on MDA-MB-231 cells were analyzed by examining the cell nucleus using DAPI staining. The results demonstrated that the treatment of FASN and ACC inhibitors could bring about the pyknosis and fragmentation of nucleus (Fig. 6e). After 24 h, the inhibitors of FASN and ACC could effectively inhibit cell migration (Fig. 6f). Above findings indicated that TVB-2640 and PF-05175157 can inhibit the proliferation and metastasis of triple-negative breast cancer MDA-MB231 cells in a concentration-dependent manner.

## Discussion

Metabolic reprogramming was identified as an important hallmark of cancer. In recent years, the metabolic reprogramming of fatty acids in tumors has attracted more and more attention. Some researchers have used MSI technology to characterize the spatial distributions of fatty acids in highly heterogeneous breast cancers, but only those high-content fatty acids such as FA-18:1, FA-20:4, FA-22:4 etc., were detected and imaged [27, 40]. In this study, by introducing BNDM as the MALDI matrix, we achieved MS imaging of 23 fatty acids in breast cancer tissues. The spatial distributions of one C_14_ FA (FA-14:1), three C_16_ FAs (FA-16:0, FA-16:1, FA-16:2), four C_18_ FAs (FA-18:0, FA-18:1, FA-18:2, FA-18:3), six C_20_ FAs (FA-20:0, FA-20:1, FA-20:2, FA-20:3, FA-20:4, FA-20:5), seven C_22_ FAs (FA-22:0, FA-22:1, FA-22:2, FA-22:3, FA-22:4, FA-22:5, FA-22:6), and two C_24_ FAs (FA-24:0, FA-24:1) were mapped. As far as we know, this is the MSI method that detects the largest number of fatty acids in biological tissues.

The spatial expressions of most fatty acids in breast cancer regions were found to be significantly higher than those in paired normal regions. By extracting the region-specific MS profiles of fatty acids in breast cancer and paired normal regions, we built a histologically authentic fatty acids reference library. Combined with multivariate statistical analysis, we further built an OPLS-DA classification model which could distinguish breast cancerous tissues from normal tissues based on the expressions of fatty acids. Without complex tissue staining and professional pathologists, we can quickly judge the tissue types by importing fatty acid MS profiles of unknown samples into the OPLS-DA classification mode. This model exhibited good performance in identifying breast cancer and normal regions with overall accuracy of 98.2%, providing a new approach for the rapid diagnosis of breast cancer.

The up-regulation of fatty acids in breast cancer tissues may be due to the metabolic reprogramming of breast cancer cells to maintain their rapid proliferation and energy metabolism. In the biosynthesis of fatty acids, palmitic acid (FA-16:0) is first produced, and then desaturated and elongated into several other types of fatty acids. ACC and FASN are two rate-limiting enzymes for the *de novo* biosynthesis of palmitic acid. In this study, by performing spatially resolved MALDI-MSI and immunofluorescence analysis, we found that the expressions of palmitic acid, ACC, and FASN in breast cancer tissues all were significantly higher than those in normal tissues. This suggests that the metabolic reprogramming of fatty acid metabolism in breast cancer occurs at both the metabolic enzyme and metabolite levels. Matching and correlating the spatial expression characteristics of metabolites and metabolic enzymes in highly heterogeneous cancer tissues will provide new insights for elucidating the complex metabolic reprogramming of cancer cells.

Targeting the altered metabolism for antitumor therapy has been extensively investigated. Inhibiting the expression of FASN in breast cancer cells can reverse the significantly up-regulated fatty acid synthesis. Actually, there is a strong interest in the development of FASN inhibitors for limiting cancer cell growth [41, 42]. Several FASN-specific inhibitors such as cerulenin [43], C75 [44], orlistat [45], and TVB-2640/3166/3664 [46–48] have been developed for cancer targeted therapy. Targeting ACC is another approach to block fatty acid synthesis and limit the growth of cancer cells [14, 49]. However, there is also a report indicating that the inhibition of ACC will reduce the oxidative stress of tumor cells and promote the growth of solid tumors [50]. In this study, we tested the effects of FASN and ACC inhibitors on triple-negative breast cancer MDA-MB-231 cells. TVB-2640 and PF-05175157 are inhibitors of FASN and ACC respectively, and have been used to treat breast cancer cells. The results suggest that both TVB-2640 and PF-05175157 can effectively constrict the cell activity, proliferation, and metastasis of MDA-MB-231 cells. Significantly, we also found that when MDA-MB-231 cells were treated with TVB-2640 and PF-05175157 in combination, the growth, proliferation, and metastasis ability of cancer cells were significantly reduced compared with those treated with TVB-2640 or PF-05175157 alone. This means that inhibiting multiple cancer metabolic targets by chemical inhibition may have a better effect on limiting the growth of cancer cells.

In summary, we developed a high-sensitive MALDI-MSI method to characterize the spatial signatures of fatty acids in heterogeneous breast cancer tissues. A total of 23 fatty acids were successfully detected and imaged. Most fatty acids exhibited higher expressions in cancer regions than those in paired normal regions. Based on the spatial expressions of fatty acids, we established an OPLS-DA classification model to identify breast cancer and normal tissues, and the overall prediction accuracy reached 98.2%. Two metabolic enzymes, ACC and FASN, participating in fatty acid *de novo* synthesis, were also found to be abnormally up-regulated in breast cancer tissues. Combining and correlating the spatial expression characteristics of fatty acids and fatty acid synthesis-related enzymes will greatly improve our understanding of breast cancer metabolic reprogramming. Furthermore, we verified the crucial role of FASN and ACC on MDA-MB-231 cells. Restraining the expressions of FASN and ACC can limit the growth, proliferation, and metastasis of triple-negative breast cancer cells, and simultaneously inhibiting the expression of these two enzymes is far superior than restraining only one of them. Overall, the results showed in this study indicate that fatty acids and their synthesis-related enzymes have undergone significant metabolic reprogramming in breast cancer, and targeting the altered fatty acid biosynthesis pathway is a potential breast cancer therapy strategy.

## Electronic supplementary material

Below is the link to the electronic supplementary material.

Supplementary Material 1

## Data Availability

All data generated and analyzed in this study are available by reasonable request of the corresponding author.
