# Supplementary material for: Imaging the metabolic reprograming of fatty acid synthesis pathway enables new diagnostic and therapeutic opportunity for breast cancer
Source: Cancer Cell Int. 2023 Apr 29;23:83. doi: 10.1186/s12935-023-02908-8 (PMC10149015; doi:10.1186/s12935-023-02908-8)
Supplement: Supplementary file 1 — Supplementary Material 1 [file 12935_2023_2908_MOESM1_ESM.docx]

**Imaging the metabolic reprograming of fatty acid synthesis** **pathway enables new diagnostic and therapeutic opportunity for breast cancer**

Fukai Wang^1,#^, Shuangshuang Ma^2,#^, Panpan Chen^3,4^, Yuhao Han^4^, Zhaoyun Liu^2^, Xinzhao Wang^1^, Chenglong Sun ^3,4,*^ and Zhiyong Yu^1,*^

^1^Breast Cancer Center, Shandong Cancer Hospital and Institute, Shandong First Medical University and Shandong Academy of Medical Sciences, Jinan 250117, China.

^2^Shandong Provincial Key Laboratory of Radiation Oncology, Cancer Research Center, Shandong Cancer Hospital and Institute, Shandong First Medical University and Shandong Academy of Medical Sciences, Jinan 250117, China.

^3^School of Pharmaceutical Sciences, Qilu University of Technology (Shandong Academy of Sciences), Jinan 250014, China.

^4^Key Laboratory for Applied Technology of Sophisticated Analytical Instruments of Shandong Province, Shandong Analysis and Test Center, Qilu University of Technology (Shandong Academy of Sciences), Jinan 250014, China.

**Table S1**. Demographic and characteristics of breast cancer patients.

| Characteristics | Number of patients (n=60) | Percentage of patients (%) |
| --- | --- | --- |
| Age(years) |  |  |
| ≤50 | 18 | 30 |
| ＞50 | 42 | 70 |
| Neoadjuvant therapy |  |  |
| YES | 10 | 17 |
| NO | 50 | 84 |
| Molecular subtypes |  |  |
| Luminal A | 16 | 27 |
| Luminal B | 26 | 43 |
| HER-2 | 5 | 8 |
| TNBC | 13 | 22 |
| Tumor size |  |  |
| Ⅰ | 34 | 57 |
| Ⅱ | 23 | 38 |
| Ⅲ | 1 | 2 |
| Ⅳ | 2 | 3 |
| Number of lymph nodes involved |  |  |
| 0(N0) | 34 | 57 |
| 1-3(N1) | 14 | 23 |
| 4-9N2) | 8 | 13 |
| ≥10(N3) | 4 | 7 |
| TNM stage |  |  |
| Ⅰ | 23 | 38 |
| Ⅱ | 24 | 40 |
| Ⅲ | 13 | 22 |


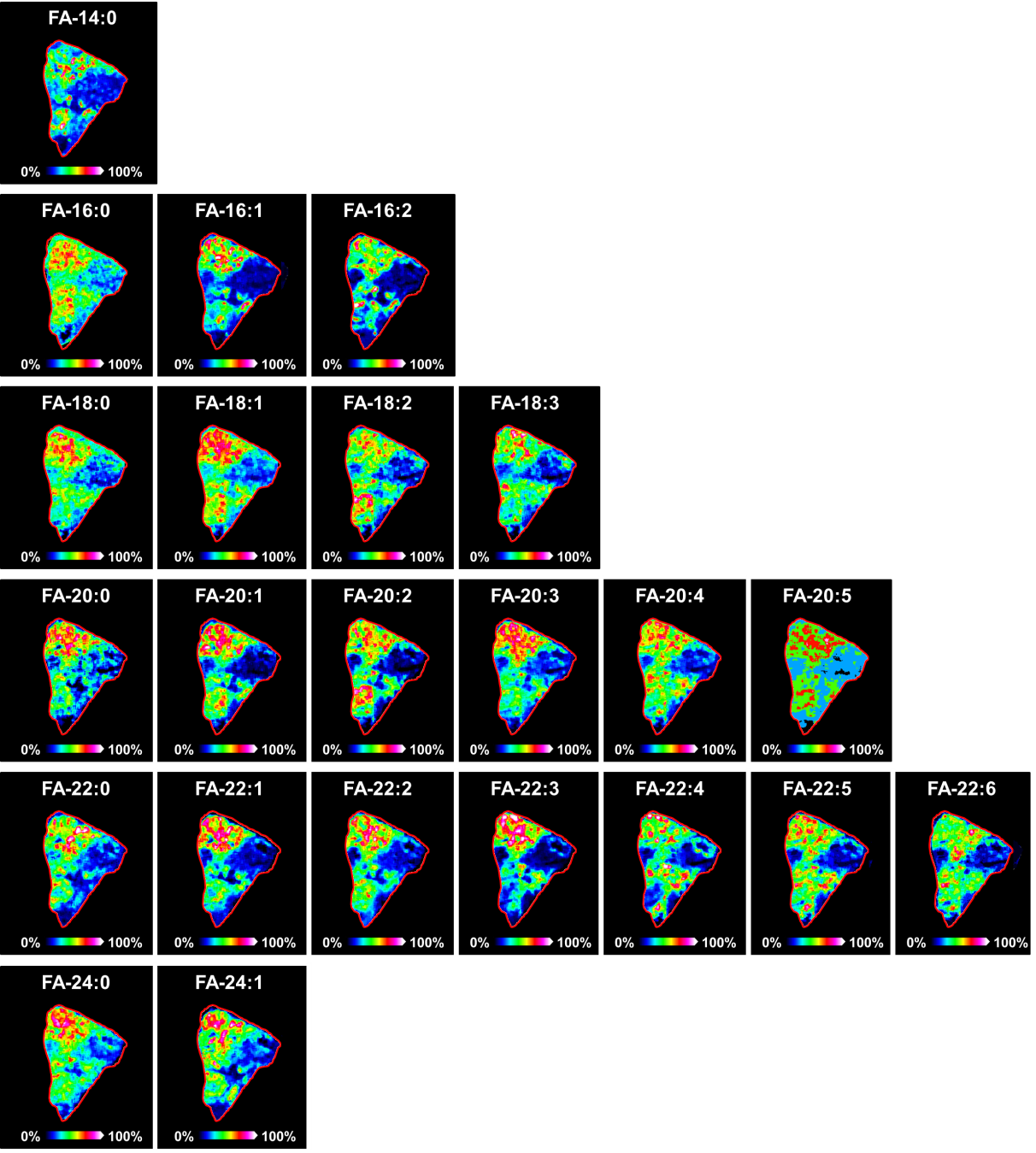


**Fig. S1**. MS images of 23 fatty acids (FA) in breast cancer tissue sections.


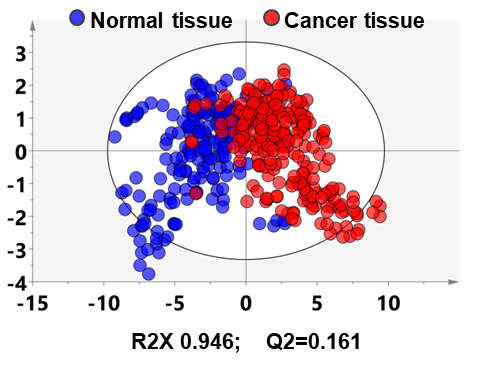


**Fig. S2**. PCA score plots based on MALDI-MSI data of 23 fatty acids in breast cancer and paired normal tissues.
